# Acute idiopathic pancreatitis is associated with more aggressive disease course in Crohn’s disease but not in ulcerative colitis

**DOI:** 10.1186/s12876-023-02790-8

**Published:** 2023-05-22

**Authors:** Karim T. Osman, Asahi Hoque, Ravi Teja Pasam, Adel Farhoud, Ahmed Abdelfattah, Vishant Ramadorai, Khadija Chaudrey, Randall Pellish

**Affiliations:** 1 Department of Internal Medicine, Lahey Hospital and Medical Center, Tufts Medical School, Burlington, 01805 MA USA; 2 Department of Gastroenterology and Hepatology, Lahey Hospital and Medical Center, Tufts Medical School, Burlington, 01805 MA USA; 3 Department of Internal Medicine, Lahey Hospital and Medical Center, Beth Israel Lahey Clinic, Burlington, 01803 MA USA

**Keywords:** Prognosis, IBD, Pancreatitis, Crohn’s disease, Extra-intestinal manifestations

## Abstract

**Purpose:**

Patients with inflammatory bowel disease (IBD), whether Crohn’s disease (CD) or ulcerative colitis (UC), have an increased risk of acute pancreatitis. The prognostic value of diagnosing acute idiopathic pancreatitis in patients with IBD is not well understood.

**Methods:**

A retrospective review of 56 patients with IBD and acute pancreatitis was conducted in a tertiary center from 2011 to 2020. Aggressive disease course was defined as (i)biologic change, (ii)biologic dose escalation, or (iii)IBD-related surgeries occurring within 1 year of acute pancreatitis diagnosis. Logistic regression modelling identified associations between covariates and an aggressive disease course.

**Results:**

Baseline characteristics between idiopathic pancreatitis and other causes of acute pancreatitis, in both CD and UC cohorts, were similar. Idiopathic pancreatitis was significantly associated with an aggressive disease course in CD (P = 0.04). No confounding factors were associated with an aggressive disease course in CD. Idiopathic pancreatitis, however, was not associated with an aggressive disease course in UC (P = 0.35).

**Conclusion:**

The diagnosis of acute idiopathic pancreatitis may provide a prognostic indicator of a more severe disease course in CD. No such association appears to exist with UC. To the best of our knowledge, this is the first study that identifies an association and possible prognostic value between idiopathic pancreatitis and a more severe disease course in CD. More studies with a larger sample size are needed to validate these findings, further define idiopathic pancreatitis as an extraintestinal manifestation of IBD and elucidate a clinical strategy to optimize care in patients with aggressive CD and idiopathic pancreatitis.

## Introduction

Inflammatory bowel disease (IBD), including both Crohn’s disease (CD) and ulcerative colitis (UC), is an idiopathic, chronic, remitting/relapsing inflammation of the gastrointestinal tract [1]. The etiology and pathogenesis remain poorly understood but is considered a multifactorial inflammatory process involving environmental factors that result in alteration of intestinal immunity and dysbiosis of gut microbiota in genetically susceptible individuals [1, 2]. Extra-intestinal manifestations (EIM) involve up to 24% of patients with IBD [3, 4].

Patients with IBD are at an increased risk of acute pancreatitis (AP) when compared to the general population. Several studies have shown a 3-4-fold increase in risk of developing AP in the setting of CD and a 2-fold risk in the setting of UC [3–5]. While most cases of AP have an identifiable etiology, a subset of cases will be defined as acute idiopathic pancreatitis [2]. Evidence indicates that acute idiopathic pancreatitis is associated with IBD [6–9]. However, no study has explored the prognostic implications of acute idiopathic pancreatitis in the setting of IBD. To address these gaps in knowledge, this study aimed to assess whether acute idiopathic pancreatitis is associated with a more severe disease course in both CD and UC.

## Materials and methods

### Patients

Using the electronic medical record, a retrospective review was conducted across a tertiary care referral center. Inclusion criteria included patients 18 years or older with established diagnoses of IBD who then developed an episode of AP between March 2011 and June 2020. Patients with recurrent AP were noted; however, only the first episode of AP was included in the analyses. Patients with chronic pancreatitis were excluded from the study. AP was diagnosed by the presence of at least two of the following: (i) acute epigastric pain; (ii) elevated serum lipase ≥ 3 times the upper normal limit; (iii) characteristic CT findings [10]. Medication-induced AP was diagnosed when there was a temporal association between the introduction of the drug and the development of AP, and when symptoms resolved after discontinuation of the offending medication [11]. Acute idiopathic pancreatitis was defined as AP after excluding other possible etiologies (gallstone, alcohol, medication, hypertriglyceridemia, iatrogenic, autoimmune, or malignancy). Recurrent AP was defined if the patient presented with a new episode of AP, as defined above, after complete resolution of symptoms. All variables were collected for the first episode of AP. EIM was defined by the presence of at least one of the following: peripheral arthritis, axial arthritis, erythema nodosum, pyoderma gangrenosum, oral ulcers, ophthalmologic complications (including episcleritis, scleritis or uveitis), and primary sclerosing cholangitis. An aggressive IBD course was defined, utilizing similar parameters described previously in the literature, as the presence of at least one of the following: (i) change in biologic therapy within 1 year of AP; (ii) biologic dose escalation within 1 year of AP; (iii) IBD-related hospitalizations or surgeries within 1 year of AP [12].

### Statistical analysis

Statistical analyses were performed using JMP Pro 14.1 (SAS Institute, Cary, NC) and R 4.0.4 (R Foundation for Statistical Computing, Vienna, Austria). Categorical data are expressed as numbers (%) and continuous variables are expressed as median (interquartile range), unless otherwise stated. Categorical and continuous variables were compared using Chi-square test and non-parametric Mann-Whitney U test, respectively. Statistical significance was determined by a P-value < 0.05. Logistic regression modeling was used to identify association between different covariates and an aggressive IBD course. Results were expressed as Odds Ratio (OR) and 95% Confidence Interval (CI). Since there were no more than 20 events in either the CD or the UC group, we could not include more than one variable in the logistic regression model [13–15].

## Results

**Fig. 1:**
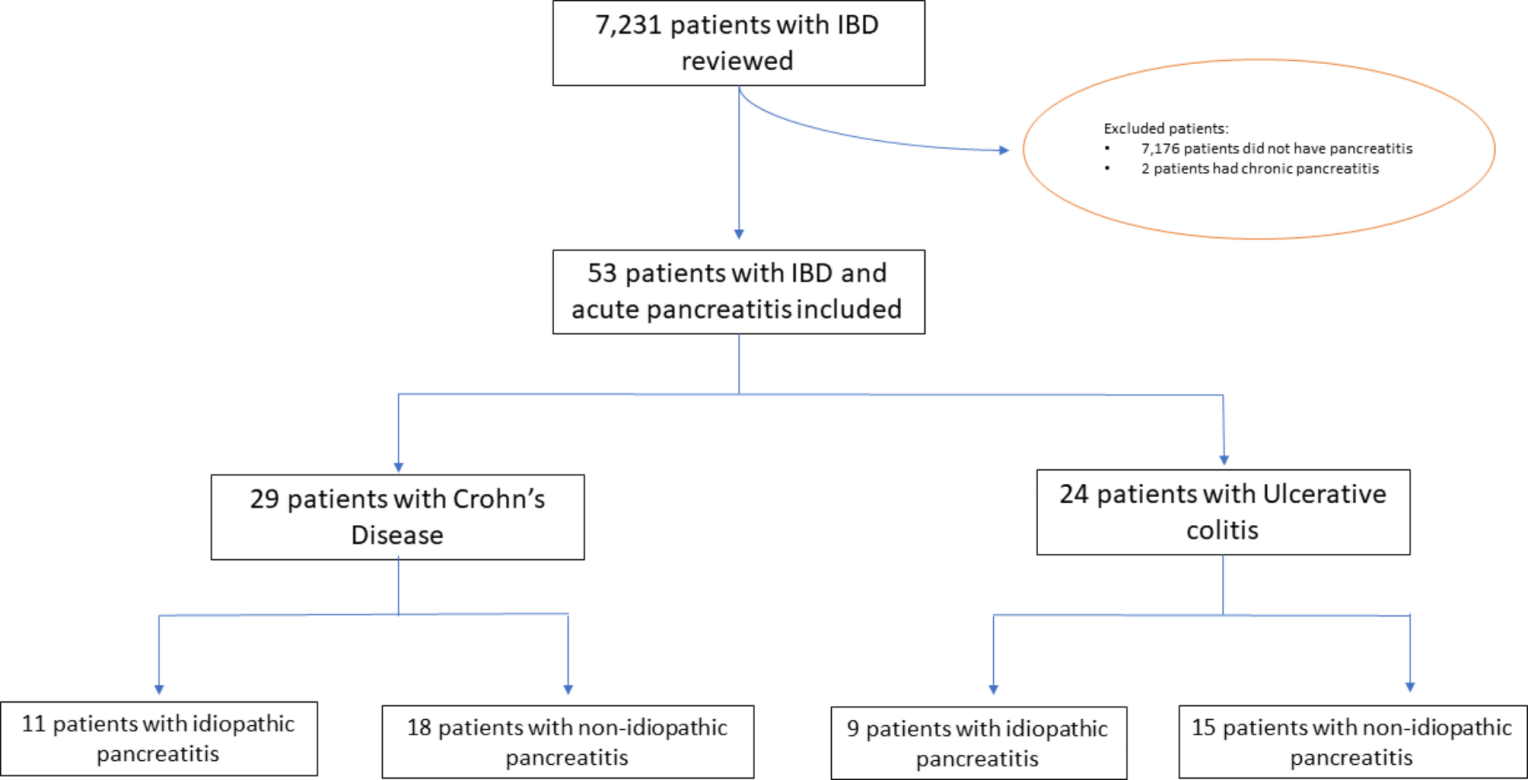
Flowchart of patient selection

After exclusion of two patients with UC due to a chronic pancreatitis diagnosis, fifty-three patients were identified with IBD and a diagnosis of AP (n CD = 29, n UC = 24). There was a total of 60 episodes of AP in those 53 patients, in a total of 7231 IBD patients identified in this tertiary care institution (cumulative incidence of AP in IBD, 0.73%). We ultimately included a total of 20 patients with idiopathic pancreatitis (11 in the CD and 9 in the UC groups) and a total of 33 patients in the comparison group (non-idiopathic pancreatitis) (Fig. 1).

**Fig. 2:**
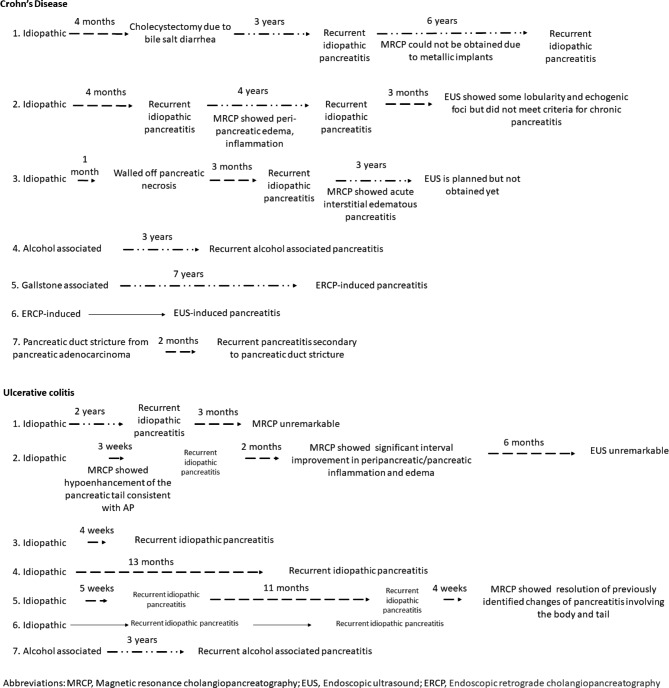
Natural course of recurrent acute pancreatitis

Baseline characteristics of patients with CD and UC are shown in Table 1. On presentation, 98.11% (52/53) of patients complained of abdominal pain, 50.94% (27/53) complained of nausea or vomiting, and 92.45% (49/53) had elevated lipase on admission. Patients were followed for a median of 2.03 (1.00-4.72) years after the diagnosis of AP. In the CD cohort, two patients had a complicated course of AP (including both idiopathic and non-idiopathic AP) – one patient had an episode of acute idiopathic pancreatitis complicated by pseudocyst and necrotizing pancreatitis, and one patient had an episode of endoscopic retrograde cholangiopancreatography (ERCP)-induced pancreatitis with pseudocyst formation. Similarly, one patient with UC with mesalamine-induced pancreatitis was complicated by pseudocyst. On follow-up, 26.42% (14/53) had recurrent AP (Fig. 2). The median duration of recurrence from the index AP was 8.50 (1.82-36) months. The median duration of recurrence of acute idiopathic pancreatitis was 3.50 (1.19–15.75) months. All patients who had recurrence of AP (including both idiopathic and non-idiopathic AP) had the same etiology as the initial episode of AP except for one patient with CD who had an episode of gallstone pancreatitis followed by an episode of ERCP-induced pancreatitis 6 years later (**Footer of** Table 1; Fig. 2). No patients died as a direct result of pancreatitis.

**Table 1:** Baseline characteristics of cohort

Variables	Crohn’s Disease (n = 29)	Ulcerative colitis (n = 24)	P-value
Duration of IBD (y)	24.51 (5.23–30.94)	15.31 (5.12–29.54)	0.64
Age (y)	51.04 (39.58–69.44)	52.02 (34.32–74.75)	0.94
Duration of hospitalization (d)	3.00 (2.00–4.00)	3.00 (2.00-4.75)	0.67
Female	17 (58.62%)	14 (58.33%)	0.98
Smoking	18 (62.07%)	11 (45.83%)	0.24
Biologics	8 (27.59%)	4 (16.67%)	0.34
Extra-intestinal manifestations ¶	13 (44.83%)	6 (25.00%)	0.13
Pancreatitis			0.47
Idiopathic pancreatitis	11 (37.93%)	9 (37.50%)	
Cholelithiasis	6 (20.69%) †	3 (12.50%)	
Alcoholic	3 (10.34%)	3 (12.50%)	
Medication ‡	2 (6.90%)	3 (12.50%)	
Hypertriglyceridemia	1 (3.45%)	1 (4.17%)	
ERCP	2 (6.90%)	4 (16.67%)	
Autoimmune	0	1 (4.17%)	
Malignancy	4 (13.79%)	0	
Recurrence of pancreatitis	7 (24.14%)	7 (29.17%)	0.68

Abbreviations: AP, Acute Pancreatitis; y, years; d, days; ERCP, Endoscopic Retrograde Cholangiopancreatography

¶ In the Crohn’s disease group, the extra-intestinal manifestations were peripheral arthritis (n = 6), axial arthritis (n = 2), both peripheral and axial arthritis (n = 1), erythema nodosum (n = 1), pyoderma gangrenosum (n = 1), and primary sclerosing cholangitis (n = 2). In the ulcerative colitis group, the extra-intestinal manifestations were peripheral arthritis (n=), and primary sclerosing cholangitis (n = 5)

† One patient in the Crohn’s Disease cohort had an episode of gallstone-induced pancreatitis and then had a subsequent episode of ERCP-induced pancreatitis

‡ Both patients who had medication-induced pancreatitis in the Crohn’s Disease cohort were due to mercaptopurine. Medication-induced pancreatitis in the Ulcerative Colitis cohort were attributed to Azathioprine, Mercaptopurine and Trimethoprim/Sulfamethoxazole.

Twenty out of the 53 patients were diagnosed with acute idiopathic pancreatitis in this cohort (n CD = 11, n UC = 9). In patients with CD, there were no statistically significant different characteristics between patients with acute idiopathic pancreatitis as compared to other causes of pancreatitis. Duration of IBD, age at the diagnosis of AP and biologic treatment were not associated with the development of different types of AP. Similarly, in patients with UC there was no statistically significant differences between patients with acute idiopathic pancreatitis as compared to other causes of AP. Patients with UC and acute idiopathic pancreatitis had a significantly higher rate of recurrent pancreatitis compared to UC patients diagnosed with other types of pancreatitis. Six patients (66.67%) with UC developed recurrent acute idiopathic pancreatitis and one patient (6.67%) developed recurrent alcoholic pancreatitis (P-value < 0.01) (Table 2). No risk factors could be identified for developing acute idiopathic pancreatitis when compared to other causes of pancreatitis (Table 3).

**Table 2:** Baseline characteristics according to the type of pancreatitis

	Crohn’s Disease (n = 29)			Ulcerative Colitis (n = 24)			Total (n = 53)		
	Idiopathic Pancreatitis (n = 11)	Other causes of pancreatitis (n = 18)	P-value	Idiopathic Pancreatitis (n = 9)	Other causes of pancreatitis (n = 15)	P-value	Idiopathic Pancreatitis (n = 20)	Other causes of pancreatitis (n = 33)	P-value
Duration of IBD (y)	26.76 (7.25–29.66)	17.95 (3.74–32.61)	0.97	9.92 (0.95–17.85)	19.01 (10.53–31.97)	0.18	11.58 (3.93–35.12)	19.01 (5.63–31.62)	0.33
Age (y)	46.29 (42.78–57.86)	56.38 (38.86–69.73)	0.61	37.25 (24.70-83.71)	52.53 (42.73–68.03)	0.77	46.00 (31.59–67.92)	52.53 (39.58–69.44)	0.48
Duration of hospitalization (d)	2.00 (1.75–2.75)	3.00 (2.00–4.00)	0.06	2.00 (2.00-5.50)	3.00 (2.00–5.00)	0.64	2.00 (2.00–4.00)	3.00 (2.00-4.25)	0.09
Female	6 (54.55%)	11 (61.11%)	0.73	7 (77.78%)	7 (46.67%)	0.13	13 (65.00%)	18 (54.55%)	0.45
Smoking	7 (63.64%)	11 (61.11%)	0.89	3 (33.33%)	8 (53.33%)	0.34	10 (50.00%)	19 (57.58%)	0.59
Biologics	3 (27.27%) ¶	5 (27.78%) †	0.98	1 (11.11%) ‡	3 (20.00%) §	0.57	4 (20.00%)	8 (24.24%)	0.72
Extra-intestinal manifestations	6 (54.55%)	7 (38.89%)	0.41	1 (11.11%)	5 (33.33%)	0.22	7 (35.00%)	12 (36.36%)	0.92
Recurrence	3 (27.27%)	4 (22.22%)	0.76	6 (66.67%)	1 (6.67%)	< 0.01	9 (45.00%)	5 (15.15%)	0.02
Number of AP episodes	1.00 (1.00–2.00)	1.00 (1.00-1.25)	0.76	2.00 (1.00–3.00)	1.00 (1.00–1.00)	< 0.01	1.00 (1.00–3.00)	1.00 (1.00–1.00)	0.02

Abbreviations: IBD, Inflammatory Bowel Disease; y, years; d, days; AP, Acute Pancreatitis

¶ Two patients were on Remicade and one patient was on Vedolizumab

† Three patients were on Adalimumab, One patient on Remicade, and one patient on 6-Mercaptopurine and Sulfasalazine

‡ One patient was on Remicade

§ Two patients were on 6-Mercaptopurine and one patient was on Azathioprine

**Table 3:** Univariable analysis of variables associated with development of acute idiopathic pancreatitis

Variables	Odds Ratio (95% Confidence Interval)	P-value
Type of IBD	1.02 (0.33–3.15)	0.97
Duration of IBD (y)	0.97 (0.92–1.02)	0.23
Age (y)	0.99 (0.96–1.02)	0.51
Age at IBD diagnosis (y)	1.00 (0.96–1.03)	0.99
Smoking	0.74 (0.24–2.26)	0.59
Female Sex	1.55 (0.50–5.05)	0.46
Biologics	0.78 (0.18–2.93)	0.71
Extra-intestinal manifestations	0.94 (0.29–2.99)	0.92

Abbreviations: IBD, Inflammatory Bowel Disease; y, years; d, days

A more aggressive IBD course was identified in 63.64% (7/11) of patients with CD and acute idiopathic pancreatitis, as compared to only 11.11% (2/18) of patients with CD and other causes of AP (OR 6.67, 95% CI 1.12–56.45; P-value 0.04). None of the other variables were associated with an aggressive disease course in CD. A more aggressive IBD course was identified in 66.7% (6/9) of patients with UC and acute idiopathic pancreatitis, as compared to only 46.67% (7/15) of patients with UC and other causes of AP (OR 2.29, 95% CI 0.43–14.31; P-value 0.35) (Table 4).

**Table 4:** Univariable analysis of variables associated with aggressive disease course

Variables	Crohn’s Disease		Ulcerative Colitis	
	Odds Ratio (95% Confidence Interval)	P-value	Odds Ratio (95% Confidence Interval)	P-value
Idiopathic pancreatitis	6.67 (1.12–56.45)	**0.04**	2.29 (0.43–14.31)	0.35
Recurrence of acute pancreatitis	0.44 (0.02–3.44)	0.49	2.81 (0.46–23.73)	0.29
Recurrence of idiopathic pancreatitis	1.67 (0.07–20.65)	0.70	6.25 (0.79-133.12)	0.13
Duration of IBD (y)	0.96 (0.87–1.03)	0.26	0.94 (0.86-1.00)	0.72
Age (y)	0.99 (0.93–1.04)	0.61	0.96 (0.91-1.00)	0.07
Duration of hospitalization (d)	0.85 (0.45–1.24)	0.50	0.75 (0.46–1.13)	0.19
Age at IBD diagnosis (y)	1.02 (0.96–1.08)	0.48	0.99 (0.94–1.04)	0.66
Smoking	5.00 (0.69-102.89)	0.17	0.25 (0.04–1.32)	0.11
Female Sex	0.43 (0.07–2.42)	0.34	0.67 (0.12–3.42)	0.63
Biologics	1.07 (0.13–6.63)	0.95	3.00 (0.32–66.60)	0.38
Extra-intestinal manifestations	1.93 (0.34–11.89)	0.46	2.00 (0.31–17.21)	0.48

Abbreviations: IBD, Inflammatory Bowel Disease; y, years; d, days

## Discussion

While there are a few studies exploring the association between IBD and AP, this cohort reflects the first focused on the association of IBD and acute idiopathic pancreatitis. These results suggest that acute idiopathic pancreatitis is associated with a more aggressive disease course in CD, but no similar association was identified with UC. Based on these results, the presence of acute idiopathic pancreatitis may represent an EIM and a prognostic factor in identifying a more aggressive disease course in CD.

Acute idiopathic pancreatitis accounts for ~ 10–35% of AP cases in the general population [16, 17]. Interestingly, acute idiopathic pancreatitis accounted for a higher percentage of AP cases in our cohort for both CD and UC (37.93% and 37.50% respectively) indicating that IBD may be a risk factor for developing acute idiopathic pancreatitis. The explanation for the association between IBD and acute idiopathic pancreatitis is unclear and may indicate that acute idiopathic pancreatitis is an EIM of IBD. Authors have speculated that pancreatitis might be an EIM of IBD [2]. AP only accounted for 0.73% of patients with IBD in our institution, however, similarly low incidence rates are found in other EIMs of IBD [18, 19]. EIM such as peripheral arthritis, erythema nodosum, and episcleritis have been associated with disease activity; however, other EIM such as uveitis or ankylosing spondylitis do not correlate with disease activity [20]. Our results demonstrate that acute idiopathic pancreatitis was associated with a more severe disease course in CD, but not in UC. However, it is not unusual for CD and UC to have different prognostic factors e.g., different studies have shown that smoking, antibiotic exposure and physical activity have varying effects on CD, compared to UC [21–23].

A possible association between acute idiopathic pancreatitis and IBD might involve a common genetic susceptibility locus between IBD and AP such as the myosin IXB (MYO9B), membrane-associated guanylate kinase inverted 2 (MAGI2) genes, and the gene encoding the partition-defective 3 protein (PARD3 gene) [24]. These genes are tight junction genes coding for scaffolding proteins that affect intestinal permeability. Alterations in the gut microbiota has been shown to be associated with IBD [25]. Such microbiota alteration may be associated with the development of pancreatitis, as noted by various studies demonstrating abnormally low levels of Bifidobacterium and Lactobacillus, as well as abnormally high levels of Enterobacteriaceae in patients with pancreatitis [26, 27].

Understanding the difference in genetic associations, microbiota dysbiosis and immune dysregulation in CD and UC is a possible key to understanding the differences noted between both diseases and might help explain the opposing association between acute idiopathic pancreatitis and the disease severity in CD and UC. An example of a possible factor that may explain the association between CD and acute idiopathic pancreatitis includes the microbiota changes of Bifidobacterium depletion in CD, that has similarly been observed in AP [23, 25, 28]. On the other hand, the previously mentioned MYO9B and PARD3 genes are more associated with UC [29, 30].

There are several limitations of this study. Firstly, it was a retrospective study conducted at a tertiary referral center. Secondly, given the rarity of the addressed disease, the sample size is small limiting its power and the generalizability of the results. This also limited the ability to conduct a multivariable analysis, as the number of variables to be included in a logistic model is dependent on the number of events [13–15]. As such, we urge the readers to interpret the results with caution. However, the cumulative incidence of AP in this cohort was similar to previously established data in the literature and thus can be considered an accurate representation of patients with AP and IBD [6, 31, 32]. In addition, the logistic regression model did not identify any other statistically significant variables, thus making it unlikely for the multi-variable analysis to have changed the results. Finally, more studies are needed to study the significance of AP, and in turn acute idiopathic pancreatitis, in an IBD cohort that consists of patients with both AP and without AP to eliminate concerns of inclusion bias.

In conclusion, this study presents data to support an association between acute idiopathic pancreatitis and IBD. The prevalence of acute idiopathic pancreatitis in the setting of IBD was noted to be higher than that in the general population. Furthermore, there is a statistically significant association between acute idiopathic pancreatitis and a more aggressive disease course in CD. Despite the lack of association between acute idiopathic pancreatitis and an aggressive disease course in UC, patients are at an increased risk of acute idiopathic pancreatitis recurrence which may contribute to increased morbidity. Thus, acute idiopathic pancreatitis might best be considered an EIM that prognosticates IBD severity. The high prevalence, association with aggressive IBD course and a possible higher recurrence rate of pancreatitis highlight a potential significant disease burden that acute idiopathic pancreatitis plays in patients with IBD.

## Data Availability

Available upon reasonable request. If access to the data is needed, please contact the corresponding author.

## References

[1] Matsuoka K, Kobayashi T, Ueno F, Matsui T, Hirai F, Inoue N (2018). Evidence-based clinical practice guidelines for inflammatory bowel disease. J Gastroenterol.

[2] Ramos LR, Sachar DB, DiMaio CJ, Colombel JF, Torres J (2016). Inflammatory bowel disease and pancreatitis: a review. J Crohn’s colitis.

[3] Blomgren KB, Sundström A, Steineck G, Genell S, Sjöstedt S, Wiholm BE (2002). A swedish case-control network for studies of drug-induced morbidity–acute pancreatitis. Eur J Clin Pharmacol.

[4] Rasmussen HH, Fonager K, Sørensen HT, Pedersen L, Dahlerup JF, Steffensen FH (1999). Risk of acute pancreatitis in patients with chronic inflammatory bowel disease. A danish 16-year nationwide follow-up study. Scand J Gastroenterol.

[5] Munk EM, Pedersen L, Floyd A, Nørgård B, Rasmussen HH, Sørensen HT (2004). Inflammatory bowel diseases, 5-aminosalicylic acid and sulfasalazine treatment and risk of acute pancreatitis: a population-based case-control study. Am J Gastroenterol.

[6] Bermejo F, Lopez-Sanroman A, Taxonera C, Gisbert JP, Pérez-Calle JL, Vera I (2008). Acute pancreatitis in inflammatory bowel disease, with special reference to azathioprine-induced pancreatitis. Aliment Pharmacol Ther.

[7] Okano A, Takakuwa H, Nishio AJIm. Idiopathic pancreatitis may be associated with ulcerative colitis. 2003;42(1):125–6. 10.2169/internalmedicine.42.125 12583634

[8] Sáez J, Martínez J, García C, Griñó P, Pérez-Mateo M (2000). Idiopathic pancreatitis associated with ulcerative colitis. Am J Gastroenterol.

[9] Seyrig JA, Jian R, Modigliani R, Golfain D, Florent C, Messing B (1985). Idiopathic pancreatitis associated with inflammatory bowel disease. Dig Dis Sci.

[10] Tenner S, Baillie J, DeWitt J, Vege SS. American College of Gastroenterology guideline: management of acute pancreatitis. The American journal of gastroenterology. 2013;108(9):1400-15; 16. 10.1038/ajg.2013.218 23896955

[11] Nitsche C, Maertin S, Scheiber J, Ritter CA, Lerch MM, Mayerle J (2012). Drug-induced pancreatitis. Curr Gastroenterol Rep.

[12] Yarur AJ, Strobel SG, Deshpande AR, Abreu MT (2011). Predictors of aggressive inflammatory bowel disease. Gastroenterol Hepatol.

[13] Concato J, Peduzzi P, Holford TR, Feinstein AR (1995). Importance of events per independent variable in proportional hazards analysis. I. background, goals, and general strategy. J Clin Epidemiol.

[14] Peduzzi P, Concato J, Feinstein AR, Holford TR (1995). Importance of events per independent variable in proportional hazards regression analysis. II. Accuracy and precision of regression estimates. J Clin Epidemiol.

[15] Peduzzi P, Concato J, Kemper E, Holford TR, Feinstein AR (1996). A simulation study of the number of events per variable in logistic regression analysis. J Clin Epidemiol.

[16] Chen Y, Zak Y, Hernandez-Boussard T, Park W, Visser BC (2013). The epidemiology of idiopathic acute pancreatitis, analysis of the nationwide inpatient sample from 1998 to 2007. Pancreas.

[17] Porges T, Shafat T, Sagy I, Schwarzfuchs D, Rahmani Tzvi-Ran I, Jotkowitz A et al. Clinical Characteristics and Prognosis of Idiopathic Acute Pancreatitis. Rambam Maimonides medical journal. 2021;12(3). 10.5041/RMMJ.10442 PMC8284986 34270401

[18] Ott C, Takses A, Obermeier F, Schnoy E, Müller M (2014). Smoking increases the risk of extraintestinal manifestations in Crohn’s disease. World J Gastroenterol.

[19] Singeap AM, Girleanu I, Diculescu M, Gheorghe L, Ciocîrlan M, Gheorghe C (2021). Risk factors for Extraintestinal Manifestations in Inflammatory Bowel Diseases - Data from the Romanian National Registry. J Gastrointest liver diseases: JGLD.

[20] Vavricka SR, Schoepfer A, Scharl M, Lakatos PL, Navarini A, Rogler G (2015). Extraintestinal Manifestations of Inflammatory Bowel Disease. Inflamm Bowel Dis.

[21] Khalili H, Ananthakrishnan AN, Konijeti GG, Liao X, Higuchi LM, Fuchs CS (2013). Physical activity and risk of inflammatory bowel disease: prospective study from the nurses. ’ Health Study cohorts.

[22] Mahid SS, Minor KS, Soto RE, Hornung CA, Galandiuk S. Smoking and inflammatory bowel disease: a meta-analysis. Mayo Clinic proceedings. 2006;81(11):1462-71. 10.4065/81.11.1462 17120402

[23] Ungaro R, Bernstein CN, Gearry R, Hviid A, Kolho KL, Kronman MP (2014). Antibiotics associated with increased risk of new-onset Crohn’s disease but not ulcerative colitis: a meta-analysis. Am J Gastroenterol.

[24] Li P, Chen K, Mao Z, Luo Y, Xue Y, Zhang Y (2020). Association between Inflammatory Bowel Disease and Pancreatitis: a PRISMA-Compliant systematic review. Gastroenterol Res Pract.

[25] Manichanh C, Borruel N, Casellas F, Guarner F (2012). The gut microbiota in IBD. Nat reviews Gastroenterol Hepatol.

[26] Akshintala VS, Talukdar R, Singh VK, Goggins M (2019). The gut Microbiome in Pancreatic Disease. Clinical gastroenterology and hepatology: the official clinical practice. J Am Gastroenterological Association.

[27] Memba R, Duggan SN, Ni Chonchubhair HM, Griffin OM, Bashir Y, O’Connor DB (2017). The potential role of gut microbiota in pancreatic disease: a systematic review. Pancreatology: official journal of the International Association of Pancreatology (IAP) [et al].

[28] Newton DF, Macfarlane S, Macfarlane GT (2013). Effects of antibiotics on bacterial species composition and metabolic activities in chemostats containing defined populations of human gut microorganisms. Antimicrob Agents Chemother.

[29] Núñez C, Oliver J, Mendoza JL, Gómez-García M, Piñero A, Taxonera C (2007). MYO9B polymorphisms in patients with inflammatory bowel disease. Gut.

[30] Wapenaar MC, Monsuur AJ, van Bodegraven AA, Weersma RK, Bevova MR, Linskens RK (2008). Associations with tight junction genes PARD3 and MAGI2 in dutch patients point to a common barrier defect for coeliac disease and ulcerative colitis. Gut.

[31] Tél B, Stubnya B, Gede N, Varjú P, Kiss Z, Márta K (2020). Inflammatory bowel Diseases elevate the risk of developing Acute Pancreatitis: a Meta-analysis. Pancreas.

[32] Weber P, Seibold F, Jenss H (1993). Acute pancreatitis in Crohn’s disease. J Clin Gastroenterol.

